# Drinking patterns, alcoholic beverage types, and esophageal cancer risk in Africa: a comprehensive systematic review and meta-analysis

**DOI:** 10.3389/fonc.2023.1310253

**Published:** 2023-12-21

**Authors:** Eugene Jamot Ndebia, Gabriel Tchuente Kamsu

**Affiliations:** Department of Human Biology, Faculty of Health Sciences, Walter Sisulu University, Mthatha, South Africa

**Keywords:** Africa, esophageal cancer, drinking patterns, alcoholic beverage types, meta-analysis

## Abstract

Africa is the continent most affected by esophageal cancer in the world. Alcoholic beverages are controversially blamed, as esophageal cancer is a rare disease in several other countries ranked in the top 10 for consumption of alcoholic beverages. This study aims to conduct a comprehensive systematic review of published literature, statistically summarizing the strength of the association between drinking patterns and types, and the risk of esophageal cancer in Africa. A computerized search of reputable databases such as Medline/PubMed, EMBASE, Web of Science, and African Journals Online was performed to identify relevant studies published up to September 2023. The quality of the studies was evaluated using the Newcastle-Ottawa scale for case-control studies and the Agency for Healthcare Research and Quality tool for cross-sectional studies. A funnel plot and Egger test were utilized to assess potential publication bias. Meta-analyses were conducted using random-effects models with RevMan 5.3 and Stata software to estimate summary effects. The systematic review identified a total of 758,203 studies, primarily from Eastern and Southern Africa. The pooled samples across all studies comprised 29,026 individuals, including 11,237 individuals with cancer and 17,789 individuals without cancer. Meta-analysis revealed a significant association between alcohol consumption and the risk of esophageal cancer (odds ratio [OR] = 1.81; 95% confidence interval [CI], 1.50-2.19). Further analysis based on the frequency of alcoholic beverage consumption indicated a stronger association with daily (OR = 2.38; 95% CI, 1.81-3.13) and weekly (OR = 1.94; 95% CI, 1.32-2.84) drinkers in contrast to occasional drinkers (OR = 1.02; 95% CI, 0.81-1.29). Additionally, consumption of traditional alcoholic beverages was significantly associated with the risk of esophageal cancer in African populations (OR = 2.00; 95% CI, 1.42-2.82). However, no relationship has been established between the exclusive consumption of non-traditional drinks and the risk of esophageal cancer. In conclusion, the results of this study confirm the hypothesis that daily and weekly drinking patterns, significantly increase the risk of esophageal cancer in Africa, while occasional consumption does not show a significant association. Additionally, the consumption of traditional alcoholic beverages is notably linked to the risk of esophageal cancer in African populations.

## 1 Introduction

Alcoholic beverages hold a significant societal role, fostering social connections and engagement, especially within specific social and religious contexts and for pleasure (1). Consumption of these beverages has witnessed a notable increase in various countries in recent years, notably since the onset of the Covid-19 pandemic (2). Particularly in Africa, alcohol consumption has surged rapidly, rising from 8% in 2018 to 15% in 2023 (3). However, excessive alcohol intake is closely linked to health risks, including mental and behavioral disorders, alcohol dependence, as well as serious noncommunicable diseases such as cirrhosis of the liver, cardiovascular diseases, and certain cancers (4). According to the World Health Organization (WHO), harmful alcohol consumption is responsible for approximately 5.3% of all global annual deaths, marking it as a significant societal issue (4). Despite governmental regulations to mitigate the adverse effects of alcoholic beverages on both the body and behavior, they remain a pivotal risk factor for numerous types of cancers, particularly esophageal cancer (5, 6).

Esophageal cancer (EC) is the seventh most common type of cancer worldwide and the sixth most common cause of cancer death (7), with an incidence rate of 3.1%. It is generally asymptomatic during the early stages of the disease. As the disease progresses, dysphagia with or without weight loss becomes apparent (8). In 2020, around 604100 new cases and 544076 deaths were recorded worldwide, of which around 40% lived along the East African corridor stretching from Ethiopia to South Africa (7). In the absence of action, 739,666 new cases and 723,466 deaths will be recorded in 2030, and 987,723 new cases and 914,304 deaths in 2040 (7). In the high-risk regions of Africa, Esophageal Squamous Cell Carcinomas are the most common type (9). The average age at diagnosis is 55 years old, and men are more affected by the disease than women (10). This disease persists as a significant obstacle for health authorities in Africa countries, especially in East African corridor.

Various researchers have independently explored the association between alcohol consumption and EC, particularly within the regions of highest incidence in Africa. However, the potential link between alcohol consumption and cancer risk in these populations remains a topic of ongoing debate, lacking a clear consensus. Studies such as those by Segal et al. (11), Middleton et al. (10), and Musukume et al. (12) have reported notably high risks (3.77 ≤ OR ≤ 5.09) of developing esophageal cancer due to alcohol consumption. Conversely, other research works like those by Leon et al. (13) and Deybasso et al. (14) have indicated lower risks (OR < 1) of esophageal cancer development among individuals who consume alcoholic beverages compared to those who abstain. While a few global meta-analyses have examined the alcohol-esophageal cancer association (6), there hasn’t been a systematic analysis focusing on Africa. Moreover, no meta-analysis to date has established the relationship between the frequency of consumption of alcoholic beverages, or the types of alcoholic beverages consumed, and EC in Africa. Above all, the rich diversity of African cultures has given rise to a multitude of traditional alcoholic beverages, the precise composition of which often remains unknown. This diversity further complicates efforts to discern the link between alcohol consumption patterns and esophageal cancer. To comprehensively understand the prevalence of esophageal cancer in Africa, we conducted a qualitative and quantitative review of the literature examining the relationship between drinking habits and this disease in these regions.

## 2 Methods

This systematic review and meta-analysis were conducted based on the guidelines of Preferred Reporting Items for Systematic Reviews and Meta-Analyses (PRISMA). The review protocol is registered at the International Prospective Register of Systematic Reviews (PROSPERO) under number CRD42023463704.

### 2.1 Eligibility criteria

The following eligibility criteria were used to identify the studies. Inclusion criteria: (1) Observational studies with Alcohol consumption as the exposure and esophageal cancer (EC) risk as the outcome. (2) All studies must contain available data reporting the relationship between alcohol drinking and EC. (3) Studies must have been conducted on the African continent (Eastern and Southern Africa region) and involve human participants. Studies were excluded according to the following exclusion criteria: (1) Unpublished articles; nonhuman research; anonymous reports; editorials, letters, commentaries, and reviews will be excluded. (2) Studies that do not provide estimates of effect in the form of odds ratios, rate ratios, risk ratios, or relative risks, or that do not allow these values to be calculated, will also be excluded. (3) Studies whose data are inaccessible, even after request to their authors, will also be excluded. (4) No sample size restrictions will be considered.

### 2.2 Data sources and search strategy

Electronic searches of databases and manual searches of other resources were conducted by two researchers (GTK and EJN) to identify published studies for review. The Medline/PubMed, EMBASE, Web of Science, and African Journals Online databases were searched for studies published up to September 2023. These searches included a mix of free text and index terms to maximize the retrieval of potentially relevant articles. The keyword combinations used by the researchers were as follows: “Alcohol” OR “Alcohol Beverage” OR “Alcoholic Beverage” OR “Alcohol Drinking” OR “Alcohol Consumption” OR “Alcohol Intake” OR “Drink Beer” OR “Drink Wine” OR “Drink Spirit” OR “Local produced Alcohol Drinking” OR “Traditional Beer” OR “Drink Kachasu” OR “Drink Busaa” OR “Drink Chang’aa” OR “Drink Gongo” OR “Risk factor” OR “Risks factors” AND “Esophageal Neoplasm” OR “Esophagus Neoplasm” OR “Esophagus Neoplasms” OR “Cancer of Esophagus” OR “Esophagus Cancer” OR “Esophagus Cancers” OR “Esophageal Cancer” OR “Esophageal Cancers” OR “Esophageal Squamous Cell Carcinoma” OR “Esophageal Neoplasms” OR “ESCC”. Searches will then be adjusted according to the requirements of each specific database (i.e. the use of operators and symbols). A manual cross-search of the references cited in the studies and the bibliographies of the documents retrieved was then carried out. No limits were established in terms of date of publication or language of publication.

### 2.3 Study selection process

The authors initiated the selection process by independently evaluating the titles and abstracts of previously identified studies. Subsequently, a second independent selection was conducted by carefully examining the full text of articles that met the initial eligibility criteria, identifying those where eligibility remained unclear. Finally, the two authors rigorously and jointly assessed the eligibility of each study, particularly those with uncertain eligibility, to determine their inclusion in the systematic review and meta-analysis. The inclusion criteria encompassed observational studies involving residents of Africa, where alcohol consumption served as either a primary or secondary risk factor for EC. At each stage of study selection, the authors worked independently, and any disparities were addressed through a consensus-seeking discussion before progressing to the next stage.

### 2.4 Data collection and quality assessment

For each study that met our eligibility criteria, comprehensive data were collected, including title, country, first author, publication date, number of cases, number of controls, participant recruitment methods, collection period, data collection methods, study population, alcoholic status, type of alcoholic beverage consumption, relative risk, and 95% confidence interval (CI), or odds ratio (OR) and 95% CI. In instances where comparative data were not available in the literature, they were calculated using appropriate statistical software. Study participants were categorized into two groups: those who had never consumed alcohol and those who had consumed alcohol. Studies presenting outcomes by race, type of beverage, or quantity smoked were aggregated before inclusion. Studies conducted across multiple countries (10) were disaggregated by country, with the author’s name duplicated and followed by the country’s initials. The authors proceeded to independently assess the quality of the studies using the Newcastle-Ottawa Scale (NOS) (15) for case-control studies and the Agency for Healthcare Research and Quality (AHRQ) tool (16) for cross-sectional studies. Any disagreements were resolved through consensus.

### 2.5 Data synthesis and analysis

In this study, participants who drink alcoholic beverages daily, weekly, or occasionally, regardless of the type are considered drinkers. Individuals who drink less than once a month are considered occasional drinkers. People who drink every weekend is classified as weekly drinker, while someone who can’t refrain from drinking every day is classified as a daily drinker. As for the types of alcoholic beverage, we have grouped alcoholic beverages into manufactured beverages (beer, whisky, and red wine) that are respected during production because of their quality standards, and into traditional beverages (busaa; Chang’aa, gongo, kachasu; Amgba; Sha’a; Tchapalo; Matango; Meloucre), that include all beverages produced by the local populations that do not meet quality standards and have no known composition. Non-drinkers were defined as individuals who abstained from alcoholic beverage consumption. For the qualitative analysis, GTK and EJN meticulously extracted qualitative data from a variety of studies and subjected them to a systematic analysis. The summarized outcomes of these analyses are presented in **Table 1**. For quantitative synthesis, statistical analyses were conducted using the RevMan 5.3 (Cochrane, USA) software for Windows. Dichotomous data relating to the association between alcoholic beverage consumption and EC were represented as odds ratios (OR) with corresponding 95% Confidence Intervals (95% CI) in a forest plot. Drinking status was utilized for stratifying the data into subgroups, and random-effects meta-analyses were performed to account for inherent differences in the study populations. Heterogeneity among the included studies was assessed using the I^2^ statistic, with significance set at P < 0.05, as described by Higgins and Thompson (43). An I^2^ value between 75% and 100% denoted substantial heterogeneity. Subgroup analysis considered the frequency of alcohol consumption by the populations to identify those demonstrating a lower risk of esophageal cancer. Differences between subgroups were evaluated through visual inspection of confidence intervals and P values. The odds ratio was employed as a measure of risk for both the subgroup and the overall association between alcohol consumption and EC. The potential small-study effects and publication bias were graphically evaluated by the funnel plot. We also conducted Egger’s test for asymmetry, where P value < 0.1 was considered significant using Stata software (Version 17.0; StataCorp). P < 0.05 (two-sided) were considered as significance level.

**Table 1:** Characteristics of the different case-control studies included for meta-analysis.

Author’s (Date)	Country	Study population	Cases/controls	Different alcoholic parameters evaluate	Period of collect	Data collection methods	Type of study
Asombang et al. (2016) (17)	Zambia	Adults (≥ 18 years)	27/45	Alcohol intake (Never alcohol use vs. ever/current alcohol use).	November 2010 and January 2012	Questionnaire	Case-control
Cunha et al. (2022) (18)	Mozambique	Adults (≥ 18 years)	143/212	Alcohol drinking (Never drinker; Ex-drinker; current drinker). Lifetime alcohol consumption (Kg ethanol). Current type of alcohol consumption (drinks/day) (Beer, Wine, Spirits, Traditional beer).	Between 2006 and 2010	Standardized questionnaire	Case-control
Dandara et al. (2005) (19)	South Africa	Adults (≥ 18 years)	272/241	Consuming alcohol (None, Alcohol consumption only, and Alcohol consumption + tobacco consumption)	Not specified	Questionnaire	Case-control
Dandara et al. (2006) (20)	South Africa	Adults (≥ 18 years)	245/288	Alcohol consumption (None, and Yes).	Between 1997 and 2003	Questionnaire	Case-control
Dessalegn et al. (2022) (21)	Ehiopia	Adults (≥ 18 years)	338/338	Ever consumed any alcoholic beverages (Yes, and No). Number of alcoholic drinks per day (Unit). Experiences of memory loss to alcohol (Yes, and No)	February 2019 to August 2020	Questionnaire	Case-control
Deybasso et al. (2022) (14)	Ethiopia	Adults (≥ 22 years)	104/208	Alcohol intake (Yes, and No).	From June 1, 2019, to June 30, 2020	Administration of questionnaire	Case-control
Geßner et al. (2021) (22)	Malawi	Adults (≥ 22 years)	157/70	Alcohol consumption (Never, Former and Current). Current type of alcohol consumption (Locally produced alcohol, Beer, Spirits).	In 2010 and between 2014–2016	Questionnaire	Case-control
Kaimila et al. (2022) (23)	Malawi	Adults (≥ 18 years)	300/300	Alcohol consumption (Never drank alcohol and Drank alcohol). Current type of alcohol consumption (Beer, Spirits, Drank Beer and Spirits, and Drank other forms of alcohol).	Between 2017 and 2020	Interviewed using a structured questionnaire	Case-control
Kayamba et al. (2015) (24)	Zambia	Adults (≥ 18 years)	50/50	Alcohol consumption (Never drank alcohol, ever drank alcohol, and currently drank alcohol).	October 2013 to May 2014	Simple questionnaire	Case-control
Kayamba et al. (2022) (25)	Zambia	Adults (≥ 18 years)	131/235	Alcohol intake (Yes, and No).	Between October 2018 and May 2021.	Interviewer-administered questionnaires	Case-control
Leon et al. (2017) (13)	Ethiopia	Adults (≥ 18 years)	73/133	Alcohol use (Never use, and ever use).	Between May 2012 and May 2013	Questionnaire	Case-control
Machoki et al. (2015) (26)	Kenya	Adults (≥ 18 years)	78/162	No alcohol history, and Alcohol history.	Between August 2008 and April 2009	Administration of the standardized questionnaire	Case-control
Masukume et al. (2022) (12)	Malawi, Tanzania	Adults (≥ 18 years)	539/593 310/313	Drank alcohol regularly (Yes, and No).	Malawi (2017-2020) and Tanzania (2015-2019)	Administration of questionnaire	Case-control
Matejcic et al. (2015) (27)	South Africa	Adults (≥ 18 years)	732/768	Alcohol drinking (Non-Drinkers, and Drinkers).	Between 2000 and 2012	Administration of the standardized questionnaire	Case-control
Menya et al. (2019) (28)	Kenya	Adults (≥ 18 years)	422/414	Alcohol consumption (Never drinker, and Drinker). Current type of alcohol consumption (Busaa, Chang’aa, Beer, and Spirits)	From 08/2013 to 03/2018	Administration of questionnaire	Case-control
Middleton et al. (2021) (10)	Kenya, Tanzania, and Malawi	Adults (≥ 18 years)	430/440 310/313 539/593	Consumed alcohol (Never, and Ever). Average ethanol intake, g per week, Type of drinker by strength of beverage, Current type of alcohol consumption (Beer, Chang’aa/Gongo/Kachasu, Spirits, Traditional beer).	Kenya: Aug 5, 2013- May 12, 2018. Tanzania: Nov 10, 2015-Dec 13, 2019. Malawi: June 1, 2017-May 24, 2020.	Administration of questionnaire	Case-control
Mlombe et al. (2015) (29)	Malawi	Adults (≥ 18 years)	96/180	Consumed alcohol (Never, and Ever).	From January 2011 to February 2013	Administration of questionnaire	Case-control
Mmbaga et al. (2020) (30)	Tanzania	Adults (≥ 18 years)	310/313	Alcohol use (Never, and Ever).	Between November 2015 and December 2019	Administration of questionnaire	Case-control
Mmbaga et al. (2021) (31)	Tanzania	Adults	471/471	Alcohol consumption (Never, Former, and Current).	Between 2013 and 2015	Administration of questionnaire	Case-control
Nhleko (2017) (32)	South Africa	Adults (≥ 18 years)	839/3557	Alcohol use (Never, and Ever).	From 1999 to 2009	Interviews with questionnaire	Case-control
Ocama et al. (2008) (33)	Uganda	Adults (≥ 18 years)	55/232	Alcohol use (Yes, and No).	From September 2004 to September 2005	Questionnaire	Cross-sectional
Okello et al. (2016) (34)	Uganda	Adults (≥ 30 years)	67/142	Alcohol use (Yes, and No).	From January 2003 to December 2014	Administration of the standardized questionnaire	Case-control
Pacella-Norman et al. (2002) (35)	South Africa	Adults (≥ 18 years)	405/2174	Alcohol consumption (Non-Drinkers, and Drinkers). Drinking frequency (Occasional drinkers, Weekly drinkers, and Frequent drinkers).	Between March 1995 to April 1999	Interviews with questionnaire	Case-control
Parkin et al. (1994) (36)	Zimbabwe	Adults (≥ 18 years)	826/3007	Alcohol consumption (Non-Drinkers, and Drinkers). Drinking frequency (Occasional drinkers, Weekly drinkers, and Daily drinkers).	Between 1963-1977	interviewed with questionnaire	Case-control
Patel et al. (2013) (37)	Kenya	Adults (≥ 18 years)	159/159	Alcohol use (Yes, and No).	Between June 2003 and July 2006	Administration of questionnaire	Case-control
Sammon (1998) (38)	South Africa	Adults (≥ 18 years)	130/130	Traditional beer use (Yes, and No).	Between 1987 - 1988	Interviews	Case-control
Segal et al. (1988) (11)	South Africa	Adults (≥ 18 years)	200/391	Traditional beer consumption (Yes, and No).	During 1984 and 1985	Interviews with questionnaire	Case-control
Sewram et al. (2016) (39)	South Africa	Adults (≥ 18 years)	670/1188	Alcohol consumption (Never, and Ever). Current type of alcohol consumption (Beer, Wine, spirits). Quantity of commercial beer consumed per week.	Between November 2001 and February 2003	Interviews with questionnaire	Case-control
Van Rensburg et al. (1985) (40)	South Africa	Adults (≥ 18 years)	211/211	Homemade spirits (Daily, Weekends, Periodically, Never).	During the period 1978-1981	Interviews were conducted by a trained African social worker.	Case-control
Vizcaino et al. (1995) (41)	Zimbabwe	Adults (≥ 18 years)	542/1705	Alcohol consumption (Non-Drinkers, and Drinkers). Drinking frequency (Occasional drinkers, Weekly drinkers, and Daily drinkers).	During the period 1963-1977.	Interviewed with a questionnaire.	Case-control
Vogelsang et al. (2012) (42)	South Africa	Adults (≥ 18 years)	550/610	Alcohol use (Yes, and No).	Between 2000 and 2010	Questionnaire	Case-control

## 3 Results

### 3.1 Summary of the characteristics of included studies

**Figure 1:**
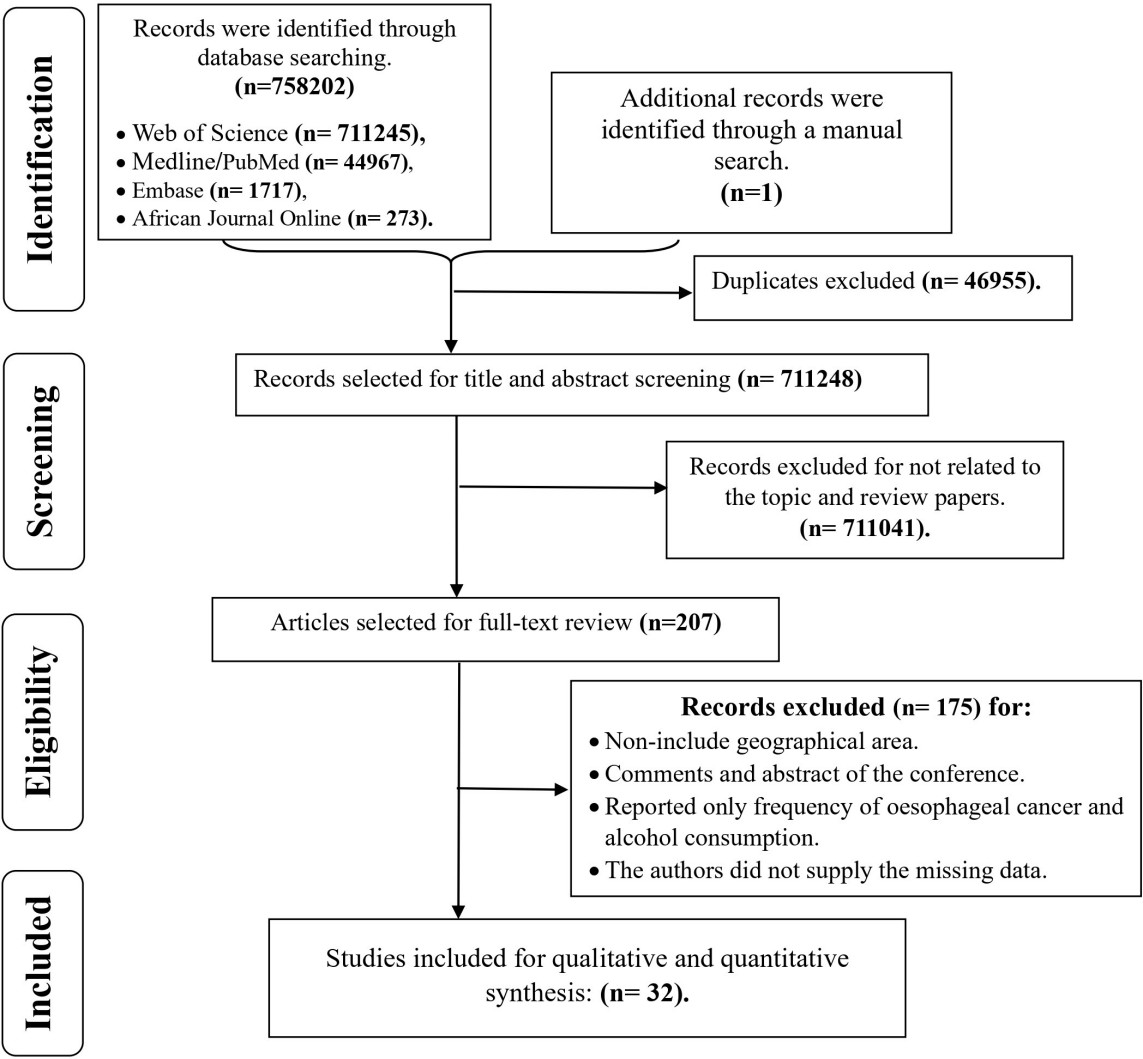
Schematical flow diagram for the selection of study included in the systematic review and meta-analysis.

The electronic and manual searches yielded a total of 758,203 studies. After eliminating duplicates (46,955 studies), a thorough review was conducted on 711,248 titles/abstracts. Following this review, 207 studies were selected for full-text examination. Subsequently, 175 studies were excluded for reasons such as non-alignment with the geographical focus of the study, being comments, abstracts from conferences, studies presenting only the frequency of cancer in alcoholics, and inadequate data even after a request to the corresponding author. Finally, 32 studies that fully met our inclusion criteria were selected for both qualitative and quantitative analysis (refer to **Figure 1**; **Table 1**).

The 32 included studies, comprising 31 case-control studies and 1 cross-sectional study, encompassed a combined sample of 29,026 individuals, consisting of 11,237 cases and 17,789 controls or non-cancer individuals. These participants were sourced from five Southern African countries (South Africa, Malawi, Mozambique, Zambia, and Zimbabwe) and four Eastern African countries (Ethiopia, Kenya, Tanzania, and Uganda) (refer to **Table 1**). The cases comprised patients diagnosed endoscopically and confirmed either histologically, through CT scans, or imaging (barium swallow) for esophageal cancer or those meeting clinical criteria for EC. The control group comprised healthy volunteers recruited from the hospital setting with no family history or affiliation with any form of cancer. The key parameters addressed in these studies included alcohol status, frequency of consumption, and the type of alcoholic beverages consumed. Data across these studies were primarily collected through questionnaires.

### 3.2 Association between drinking alcohol and esophageal cancer risk

**Figure 2:**
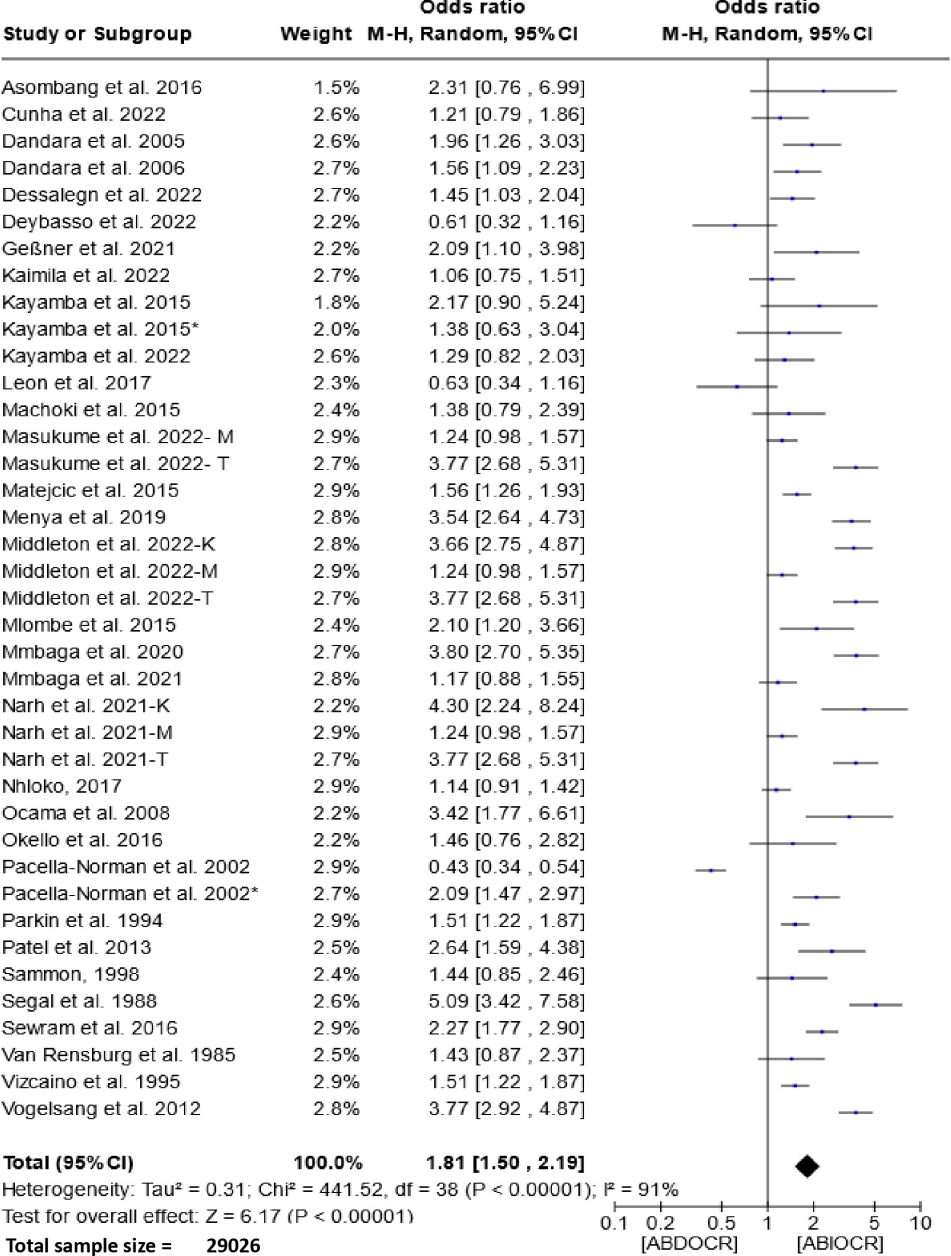
Forest plots for the association between drinking alcohol and esophageal cancer risks. Data is presented as Odds Ratio with 95% Confidence Intervals (CI) utilizing a random-effects model. Heterogeneity among the studies was assessed using the l^2^ statistic with a significance level of P < 0.05. ABDOCR, Alcoholic beverages decrease esophageal cancer risk; ABIOCR, Alcoholic beverages increase esophageal cancer risk.

The forest plot presented in **Figure 2** illustrates the association between the consumption of alcoholic beverages and esophageal cancer in East and Southern Africa. Analysis of this figure unveils a pooled odds ratio of 1.81 (95% CI, 1.50-2.19, P < 0.00001) and a substantial degree of heterogeneity (I^2^ = 91%). These findings strongly suggest a significant association between alcohol consumption and esophageal cancer.

### 3.3 Assessment of publication bias

**Figure 3:**
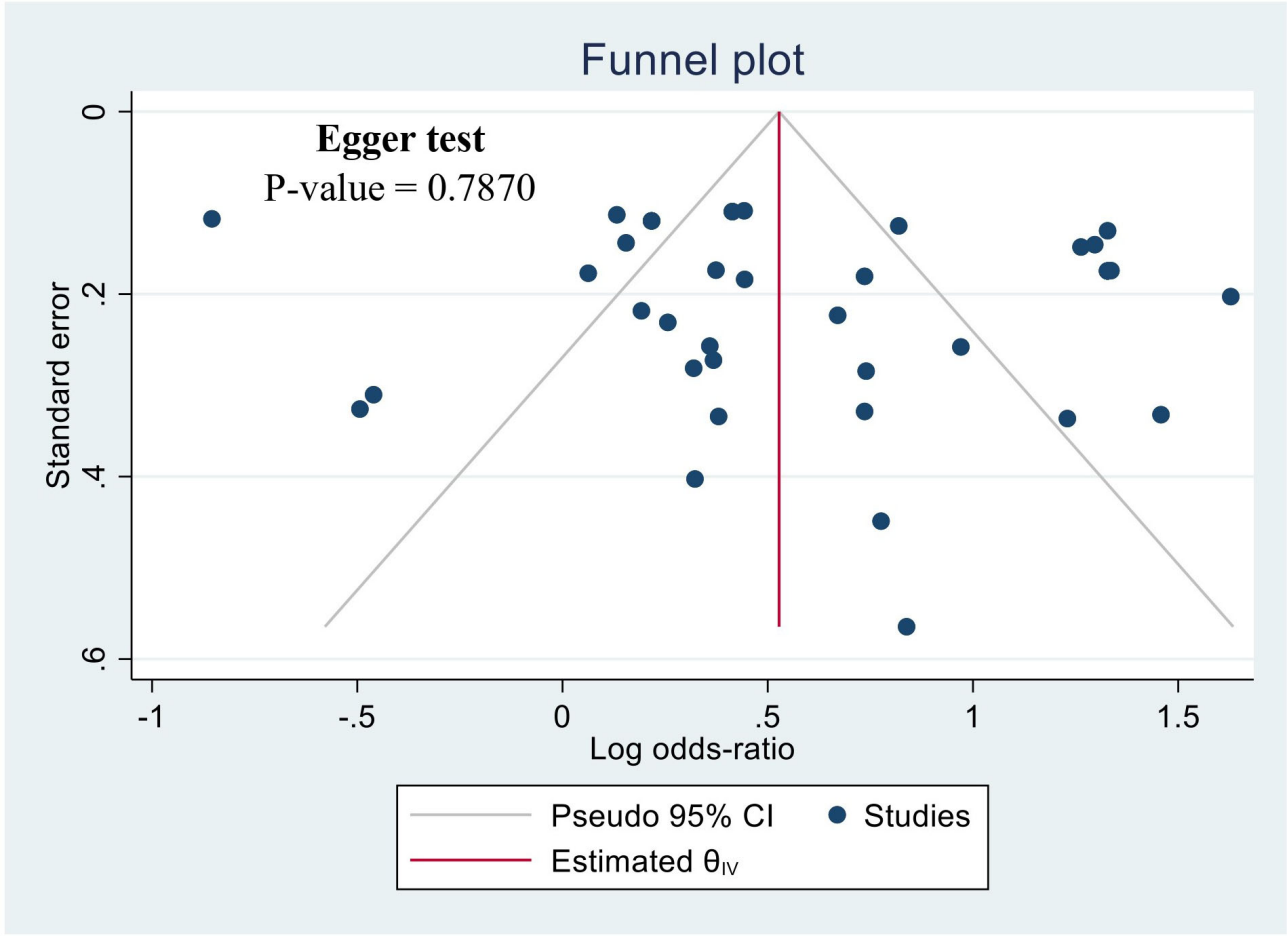
Funnel plot of the studies based on the association between drunk alcoholic beverages and the risk of esophageal cancer.

The potential for publication bias was assessed using a funnel plot illustrated in **Figure 3**. Visual inspection of the funnel plot did not provide evidence for asymmetry. The Egger regression test also did not detect a potential publication bias (P-value = 0.7870). likewise, it is noteworthy that the sensitivity analysis, excluding these studies individually did not alter the significance of the overall outcome.

### 3.4 Effect of drinking frequency on etiology of esophageal cancer

**Figure 4:**
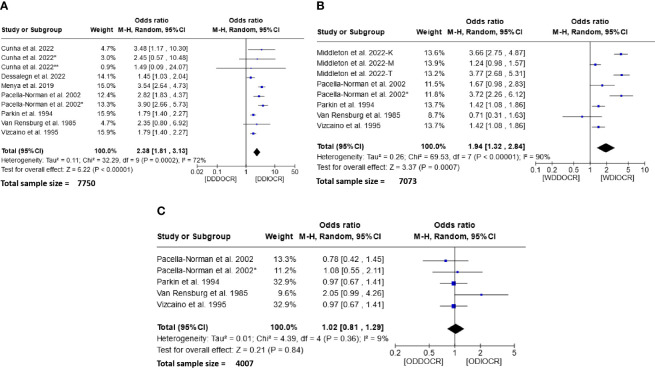
Forest plots for the association between Frequency of drinking alcohol and esophageal cancer risks. Data is presented as Odds Ratio with 95% Confidence Intervals (CI) utilizing a random-effects model. Heterogeneity among the studies was assessed using the I^2^ statistic with a significance level of P < 0.05. **(A)** Daialy alcohol drinker; **(B)** Weekly alcohol drinker; **(C)** Occasionally alcohol drinker; DDDOCR, daily drink decreases esophageal cancer risk; DDIOCR, daily drink increases esophageal cancer risk; WDDOCR, weekly drink decreases esophageal cancer risk; WDIOCR, weekly drink increases esophageal cancer risk; ODDOCR, Occasionally drink decrease esophageal cancer risk; ODIOCR, Occasionally drink increase esophageal cancer risk.

The meta-analysis of data concerning the influence of the frequency of alcoholic beverage consumption on the etiology of esophageal cancer is depicted in **Figure 4**. Analysis of this figure revealed that both daily (**Figure 4A**) and weekly (**Figure 4B**) alcohol consumption significantly increased the risk of esophageal cancer. The risk was notably higher in daily drinkers [OR = 2.38 (95% CI, 1.81-3.13); I^2^ = 72%, and P < 0.00001] than in weekly drinkers [OR = 1.94 (95% CI, 1.32-2.84); I^2^ = 90%, and P=0.00007]. However, no statistical significance was observed in the occasional drinker subgroup, with an OR of 1.02 (95% CI, 0.81-1.29), P=0.84, and a minimal degree of heterogeneity (I^2^ = 9%) (**Figure 4C**).

### 3.5 Effect of traditional alcoholic beverages on etiology of esophageal cancer

**Figure 5:**
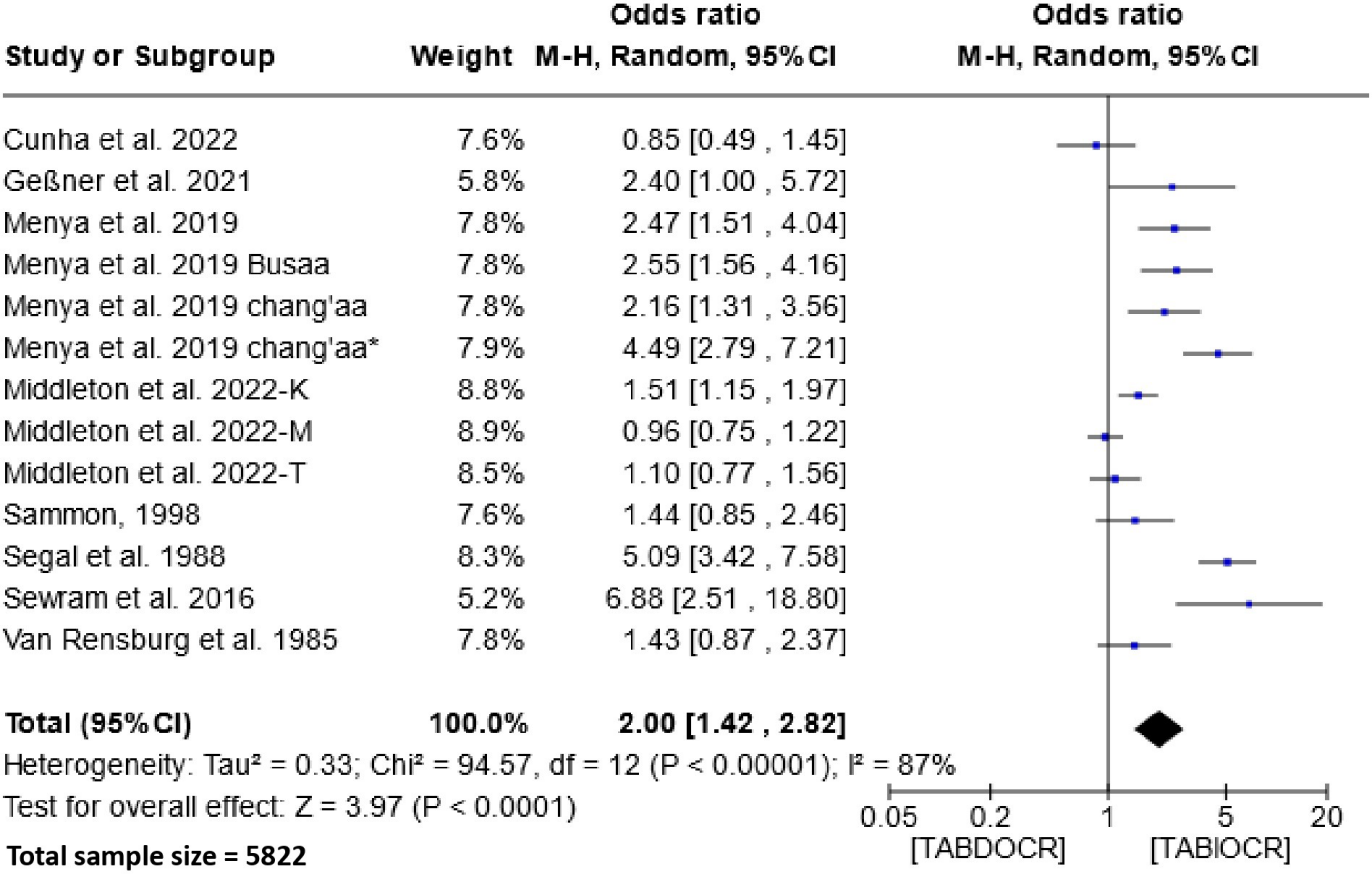
Forest plots for the association between traditional alcoholic beverages and esophageal cancer risks. Data is presented as Odds Ratio with 95% Confidence Intervals (CI) utilizing a random-effects model. Heterogeneity among the studies was assessed using the I^2^ statistic with a significance level of P < 0.05. TABDOCR, Traditional alcoholic beverage decreases esophageal cancer risk; TABIOCR, Traditional alcoholic beverages increase esophageal cancer risk.

The impact of traditional alcoholic beverage consumption on the etiology of esophageal cancer is depicted in **Figure 5**. Analysis of this figure demonstrates a significant association between the consumption of these beverages and the risk of esophageal cancer within the populations of Southern and Eastern Africa. The Odds Ratio (OR) for this association is 2.00 (95% CI, 1.42-2.82), with a heterogeneity of 87% and P < 0.00001.

### 3.6 Effect of non-traditional alcoholic beverages on etiology of esophageal cancer

**Figure 6:**
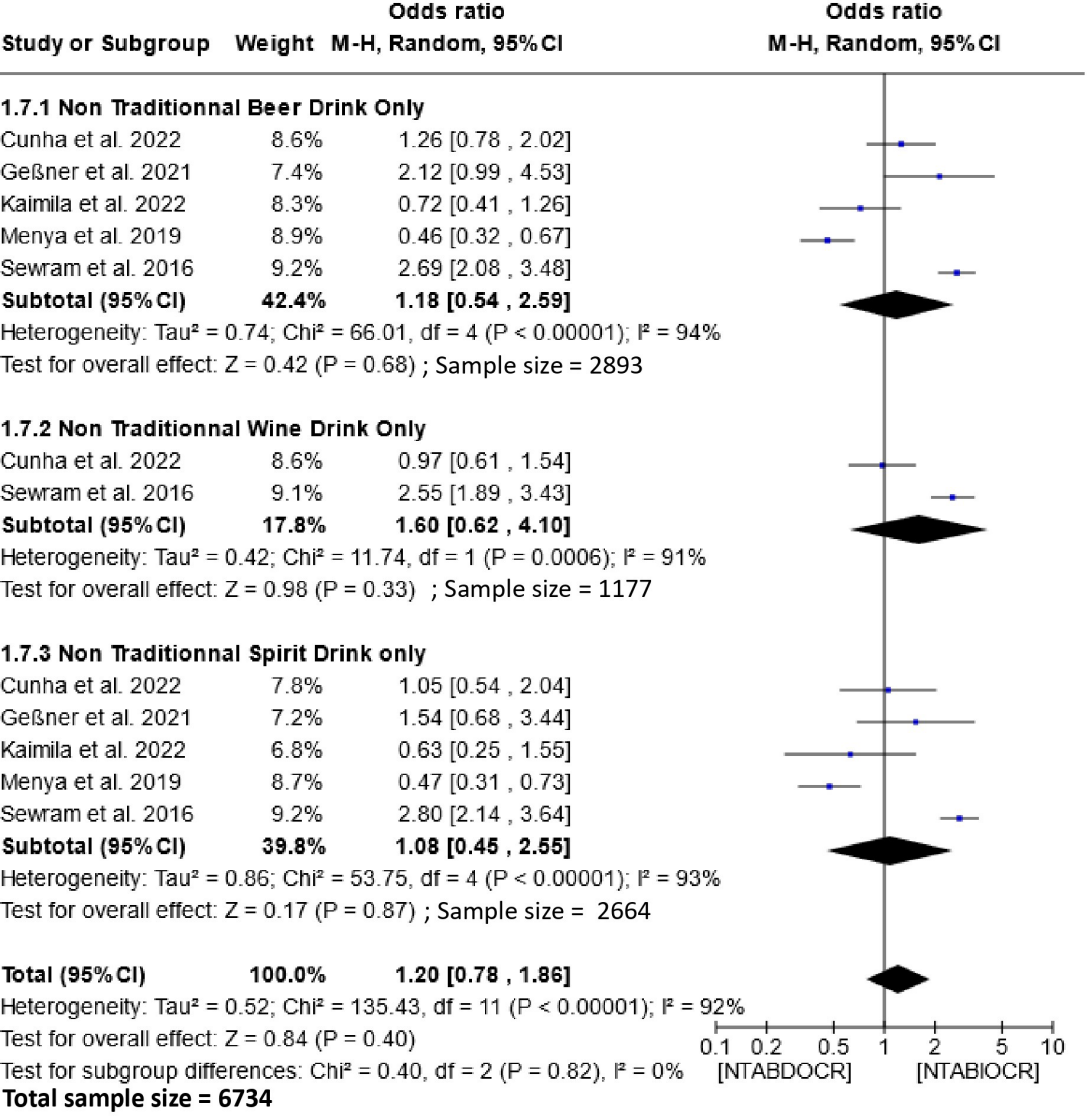
Forest plots for the association between non-traditional alcoholic beverages and esophageal cancer risks. Data is presented as Odds Ratio with 95% Confidence Intervals (CI) utilizing a random-effects model. Heterogeneity among the studies was assessed using the l^2^ statistic with a significance level of P < 0.05. NTABDOCR, Non-traditional alcoholic beverage decreases esophageal cancer risk; NTABIOCR, Non-traditional alcoholic beverages increase esophageal cancer risk.

The impact of non-traditional alcoholic beverage consumption on the etiology of EC is depicted in **Figure 6**. Analysis of this figure demonstrates a non-significant association between the consumption of these beverages and the risk of esophageal cancer within the populations. The Odds Ratio (OR) for this association is 1.20 (95% CI, 0.78-1.86), with a heterogeneity of 92% and P =0.82. Subgroup analysis showed no significant association between exclusive consumption of non-traditional beers [1.18 (95% CI, 0.54-2.59); P=0.68], wines [1.60 (95% CI, 0.62-4.10); P=0.33] and spirits [1.08 (95% CI, 0.45-2.55); P=0.87] and the risk of esophageal cancer.

## 4 Discussion

Alcohol consumption is deeply ingrained in the social fabric of African communities, both rural and urban, predating colonization (44). Celebratory events involve the consumption of alcoholic beverages, often pursued for pleasure (1, 44, 45). However, this widespread practice is a major contributor to global morbidity and mortality, notably linked to chronic health issues such as esophageal cancer (46–48).

This systematic review and meta-analysis focused on evaluating the relationship between alcoholic beverage consumption and the risk of esophageal cancer in Africa. Despite ethanol, the primary component of alcoholic drinks, not being inherently carcinogenic (6), our meta-analysis revealed a significant association between alcoholic beverage consumption and esophageal cancer risk (OR = 1.81; 95% CI, 1.50-2.19) across 32 studies solely from Southern and Eastern Africa.

The robust link between alcoholic beverage consumption and esophageal cancer risk is primarily attributed to the first metabolite, acetaldehyde, formed during ethanol oxidation by Acetaldehyde dehydrogenases. Acetaldehyde, with its genotoxic effects on cellular processes, can bind to DNA or react with various cellular residues, causing DNA oxidation and lipid peroxidation (49–51). Genetic polymorphisms in ethanol metabolism enzymes and a mismatch in alcohol dehydrogenase (ADH) and aldehyde dehydrogenase (ALDH) activities also contribute to alcohol-induced neoplasms (52, 53).

Our meta-analysis further highlighted a significant association between frequent alcohol consumption (daily [OR = 2.38 (95% CI, 1.81-3.13)] and weekly [OR =1.94 (95% CI, 1.32-2.84)] and increased EC risk, while occasional consumption [OR =1.02 (95% CI, 0.81-1.29)] showed no significant association. This suggests that repeated alcohol intake may exacerbate carcinogenic effects on the esophagus. The accelerated division of esophageal stem cells due to cytotoxic ethanol concentrations, as seen in regular alcohol consumption, may play a role in maintaining cellular homeostasis challenged by carcinogens from ethanol (54, 55).

Moreover, our study demonstrated a substantial association (OR = 2.00; 95% CI, 1.42-2.82) between traditional alcoholic beverage consumption and EC risk in Southern and Eastern African populations. This association is potentially influenced by the uncontrolled ethanol content and the presence of carcinogenic substances like methanol, formaldehyde, and formic acid in traditional beverages (56–59). Additionally, these beverages might contain other carcinogens like aflatoxin, lead, and nitrosamines (28). However, the non-significance observed with non-traditional alcoholic beverages reflects the strict control of the various ingredients contained in the products and above all the absence of methanol. Potential toxins are destroyed in the beverages during pasteurization (60). Hence, EC in these people may be due to other risk factors.

In the present study, global comparisons and certain analyses of subgroups revealed a strong heterogeneity among studies. We have observed that the frequency of consumption (daily and weekly) and types of alcoholic beverages drinks could significantly influence the heterogeneity among the included studies. Obviously, variations in alcohol concentrations and other potential substances contained in different beverages can affect the results. Additionally, the high heterogeneity could be attributed to variation in population characteristics, like comorbidities, cancer stage, lifestyle (smoking, dietary habits, and other), and socioeconomic status (61). Furthermore, variation can be attributed to study characteristics, such as outcome measurement and study design (62).

Considering these findings, it is imperative that Southern and Eastern African governments formulate and implement consistent public education policies on the adverse health effects of frequent alcoholic beverage consumption, especially traditional ones. Urgent measures are needed to regulate the sale of questionable origin drinks and those failing to comply with international standards, containing numerous carcinogenic substances like methanol.

This study underscores the pivotal role of alcoholic beverage consumption, especially at regular intervals, in the incidence of EC in major African regions. It advocates for immediate governmental action to educate the public and enact regulations to mitigate these significant public health concerns.

## 5 Limitations

This study is subject to several limitations. Firstly, observational studies inherently carry a risk of confounding and bias, and the potential for recall and selection biases was notable in this meta-analysis. Secondly, available data focused on Eastern and Southern African countries, data availability was then limited, with only nine countries contributing studies, and not all regions within these countries were adequately covered, affecting the comprehensiveness of the assessment. The lack of data on the quantity of alcoholic beverages and ethanol consumed hindered a deeper understanding of the association between consumption levels and EC risk. Another major limitation of this work is the absence of data in the included literature that could be used to verify whether age plays an important role in this association between alcoholic beverage consumption and EC risk. Addressing these limitations through future studies would reinforce the robustness of the association between alcohol consumption and EC risk.

## 6 Conclusion

In summary, this systematic review and meta-analysis establish a significant link between alcoholic beverage consumption and the heightened risk of EC in Africa. The risk amplifies with increased frequency of consumption, highlighting the urgency for targeted public health interventions. Additionally, traditional alcoholic beverages emerge as significant contributors to EC risk. Regions facing elevated risks should formulate and enact comprehensive strategies to educate the populace about the perils of alcohol misuse. A concerted subregional effort is imperative to guide policymakers in countering the proliferation of substandard and counterfeit beverages and to mitigate the health hazards posed by alcohol. Future research endeavors should prioritize determining ethanol concentrations associated with esophageal cancer risk in the African context to further inform prevention and intervention strategies.

## Author contributions

GK: Conceptualization, Data curation, Formal analysis, Investigation, Methodology, Software, Validation, Writing – original draft, Writing – review & editing. EJ: Conceptualization, Data curation, Formal analysis, Investigation, Methodology, Project administration, Software, Supervision, Validation, Writing – review & editing.
